# Effect of ribs in a suddenly expanded flow at sonic Mach number

**DOI:** 10.1016/j.heliyon.2024.e30313

**Published:** 2024-04-26

**Authors:** Ambareen Khan, Sher Afghan Khan, Vijayanandh Raja, Abdul Aabid, Muneer Baig

**Affiliations:** a School of Aerospace Engineering, Universiti Sains Malaysia, Penang, Malaysia; b Department of Mechanical and Aerospace Engineering, Kulliyah of Engineering, International Islamic University Malaysia, Kuala Lumpur, Malaysia; c Department of Aeronautical Engineering, Kumaraguru College of Technology, Coimbatore, 641049, Tamil Nadu, India; d Division of Research and Development, Lovely Professional University, Phagwara, Punjab, 144401, India; e Department of Engineering Management, College of Engineering, Prince Sultan University, PO BOX 66833, Riyadh, 11586, Saudi Arabia

**Keywords:** Base pressure, Flow characteristics, Nozzle pressure ratio, Suddenly expanded flow, Rib geometry

## Abstract

This study aims to assess the influence of a rib on the base pressure and the flow development in an abruptly expanded duct at sonic Mach number. Initially, the simulations were done to validate the experimental results, keeping all the parameters the same. Accordingly, a duct-of-area ratio of 6.25 was considered for validation. Five ribs of aspect ratios 3:1, 3:2, and 3:3 were used as a first step, and simulations were performed for the same nozzle pressure ratios. Results indicate that for an area ratio of 6.25, there is a continuous decrease in the base pressure despite the nozzles being highly under-expanded. The lower aspect ratio of the rib tends to reduce the base pressure, whereas a higher aspect ratio effectively increases the base pressure for an area ratio of 6.25. Later simulations considered a single rib instead of five ribs, varying the rib's heights from 1 mm to 5 mm. Results show that the base pressure increases considerably when rib heights are 4 mm and 5 mm. The influence of ribs at two duct diameters (25 mm and 18 mm) is studied to assess the impact of a decrease in the area ratio and, hence, a decrease in the relief available to the flow. Results of duct 18 mm show that passive control becomes very effective when a rib of 3 mm height is located at a 3D position. The differences in the base pressure, velocity, and pressure field for each case are explored. The simulation results indicate that the rib breaks the primary vortex at the base and forms multiple vortices. Turbulent kinetic energy increases in the presence of ribs more than without a rib.

## Nomenclature

*P*_*0*_: stagnation pressure (Pa)
*P*_*a*_: atmospheric pressure (Pa)
*D*: diameter duct (mm)
L: duct length
*NPR*: nozzle pressure ratio
*W*: width (mm)
*H*: height (mm)
*ρ*_*a*_: density (kg/m^3^)
*u*_*d*_: axial 2-momentum velocity (m/s)
*v*_*a*_: radial r-momentum velocity (m/s)
*T*_*a*_: static temperature (K)
*μ´*: viscosity
*μ*_*0*_*´*: reference viscosity
*T*_*0*_: reference temperature (K)
*S´*: Sutherland constant
*k*: thermal conductivity (W/m^2^K)
*σ*_*k*_: turbulent Prandtl number
*ε*: turbulent kinetic energy
*M*_*X*_: turbulence generation
TKE: turbulence kinetic energy

## 1 Introduction

In many different flow systems, the sudden increase in the area of the duct is an issue of broad interest. Utilizing the low base pressure that arises from the abrupt relaxation of the shear layer from the intake route at the entry to the sudden enlargement, the enlarged duct is often smooth and continuous inside. The vortex dynamics created by the abrupt expansion of the flow in the larger duct determine the base pressure and the flow field downstream of the base. However, this work focuses on air jets that abruptly grow into a larger duct with annular ribs before the jet is exhausted into the atmosphere. In the current study, the primary vortices caused by the free expansion of the shear layer at the base and the secondary vortices created by the ribs in the larger duct work together to produce the base pressure and the growth of the flow field downstream of the base zone. There is a discussion of a few works that have a direct bearing on the current investigation.

At transonic Mach number contribution from the base, drag is 60 % of the total vehicle drag. This contribution is due to the low-pressure region formed at the base [1], and increasing the base pressure reduces the base drag [2]. The base pressure can be controlled by using active and passive control approaches. Applying blowing or suction methods is used in dynamic control, requiring an expensive control mechanism. In some cases, additional external energy sources are impossible to acquire. By contrast, passive control does not require any external energy source. Instead, it controls the base pressure according to the geometrical changes in the flow field using ribs, cavities, boat tails, splitter plats, and locked vortex mechanisms [3]. The jet-pump theory is usually employed using passive control when controlling the base pressure [4]. This theory defines the boundary layer as the fluid source at the corner. It can be treated as the source of two fluid masses: the boundary layer around the corner (recirculation zone) and the backflow along the wall in the expanded section. In the recirculation zone, the pressure at the edge of the base in subsonic flows is equal to the converging nozzle's exit pressure. The sudden expansion flow field, where the shear layer meets the duct wall, is called the reattachment point. The shear layer that meets at the reattachment point acts as the diving streamline.

Due to the delayed transition, the suction is applied through the boundary layer to reduce drag in fully submerged 3D bodies [5]. Many factors can influence the fluctuation in base pressure. The enlarged pipe's base pressure depends on the area ratio, L/D ratio, stagnation pressure in the main settling chamber, and inertia level at the exit of the nozzle. Furthermore, one can identify the duct's L/D ratio, which yields the maximum pressure at the exit plane for a given area, and nozzle pressure ratios. At the same time, one can find the combination of parameters resulting in a minimum pressure loss. Separating the shear layer and the reattachment point with an enlarged duct depends on the area ratio, nozzle pressure ratio, and the Mach number [6].

Dimples and cavities are also reported to control the base pressure at various duct lengths, given that the wall pressure is not adversely affected by the dimples [7]. Rathakrishnan et al. investigated the use of cavities on a suddenly expanded duct at subsonic Mach numbers [8]. They found that these cavities minimize the base pressure, given their aspect ratios ranging from 2 to 3. However, a cavity with an aspect ratio of 3–4 demonstrated a higher base pressure. In other studies, a 50 % increment in base pressure and a 5 % reduction in total drag was due to the base cavities, especially at a Mach number of 2 [9]. The base pressure coefficient increased the shedding frequency by 4 %–6 %, showing an insignificant effect on the vortex structure [10]. At a sonic Mach number, a minimum base pressure was observed as the wall pressure distribution became smoother due to the ribs’ presence with an aspect ratio of 3:1. However, the base pressure increases with the increment of the 3:1 aspect ratio of the rib to 3:3 [11].

Ambareen et al. [12] investigated experimentally using a passive control technique. They visualized the free jet flow characteristics from the converging nozzle using a shadow graph. They performed a computational fluid dynamics (CFD) analysis to analyze the pressure distribution when the rib positions varied from 1D to 4D through a converging nozzle at a sonic Mach number, the area ratio of 4, and the rib aspect ratios of 3:1 and 3:3. The variation in rib positions influenced the distance among successive compressed zones. The compression zone's effect diminished as the flow traveled longitudinally, reducing the length among the compressed zones. Besides, the strength of the zones increased along with rib height. Reduced base pressure was observed when the rib aspect ratio was increased from 3:1 to 3:3. The base pressure and velocity decreased as the rib location changed from 2D to 4D. Different NPRs saw an increase in the base pressure, as well. Due to the rapid flow expansion, a high increment in base pressure was observed at rib aspect ratios of 3:2 and 3:3, especially at an NPR higher than 2.5.

Nozzle investigation in terms of design always found a critical study when it is used for supersonic flow control [13]. Base pressure in sudden expansion ducts is one of the foremost problems in creating drag inside the duct of the nozzle, and therefore, control of base pressure is essential [14,15]. Performed a simulation and experiment in their study of ribs' role in controlling base pressure by Ambareren et al. [16]. However, they only focused on a 28 mm square duct with an NPR ranging from 2 to 8, a Mach number ranging from 1 to 2.5, and a fixed L/D ratio. They discovered that the base pressure continued to fall as the NPR increased despite the jets being under-expanded due to the enlarged duct's large area ratio. Khan and Aabid [17] also performed a CFD analysis to observe the flow characteristics influencing base pressure changes. They considered a convergent-divergent nozzle with a supersonic Mach number of 1.87. They applied the active control approach by using microjets. Four microjets with an orifice diameter of 1 mm were located at the divergent nozzle exit. They varied from 1 to 10 as the ratio of L/D and nozzle was operated at an NPR ranging from 3 to 11. They selected the k-epsilon standard wall function to represent the turbulence inside the nozzle. In another study, the author utilized cavity-based control to manage base pressure variations at supersonic Mach numbers, employing the same CFD approach and varying parameters to enhance existing results [18]. Subsequently, the same authors explored variations in nozzle design to investigate flow control effectiveness [19]. Several studies employing the CFD method have focused on the effect of expansion level and relief on the shear layer in a suddenly expanded flow within a nozzle's sudden expansion duct [20].

Supersonic flow control has consistently been a subject of advanced research. Notably, the nozzle is considered crucial for supersonic flow control, and its modification over the years is evident in review articles [21,22]. However, these reviews primarily focus on either passive or active control of flows. Additionally, flow control can be achieved by optimizing the shape of the nozzle into an extendable format. In some cases, work by Faccioli et al. [23] involves the creation of nozzle structures with a central body aimed at flow separation. In another study, flow manipulation was achieved using grooved tabs within the nozzle [24]. While these works primarily focus on controlling flow inside the nozzle, research by Afkhami and Fouladi [25] aimed to optimize a supersonic exhaust diffuser's starting and terminating phases with a conical nozzle to increase exit flow. Another study used turbulence models to mix supersonic flow in an air intake [26].

Given the limitations of computational work [27], this study evaluates the effect of different rib designs and sizes using CFD and a standard turbulence model to understand the flow behavior inside the converging nozzle. This study focuses on passive control by considering rib location, rib aspect ratio, and duct area ratio in controlling the base pressure in a sonic Mach flow. The CFD simulation results are validated based on Rathakrishnan's findings [11]. This paper considers ribs at 1D (diameter of the duct) to 4D positions with aspect ratios of 3:1 to 3:5, NPRs of 1.5–5, and duct diameters of 25 mm and 18 mm. This study also examines rib location, rib aspect ratio, nozzle pressure ratio, and area ratio on base pressure.

## 2 Overview of the test case

The suddenly expanded flow setup consists of a converging nozzle operating at a sonic Mach number. The flow from the stagnation point has a pressure, P_0_, that converges slowly from the nozzle's inlet. The atmospheric pressure, P_a,_ is lower than the stagnation pressure, thereby expanding the flow from the nozzle through a 25-mm-diameter duct attached at the end of the nozzle. The flow separates and recirculates at the nozzle due to low base pressure. In this work, rectangular ribs with a width, W, and height, as well as H of the rectangular cross-section, are used as the passive control mechanism. The influence of ribs on NPR is analyzed by varying the P_o_/P_a_ ratio from 1.5 to 5. The analysis is then extended by placing the rib at 1D (D = 25 mm), 2D, 3D, and 4D positions. The aspect ratio of the rib (W: H) is set from 3:1 to 3:5. Two duct diameters (25 mm and 18 mm) are considered in analyzing the influence of the rib on base pressure due to variation in duct area.

**Fig. 1:**
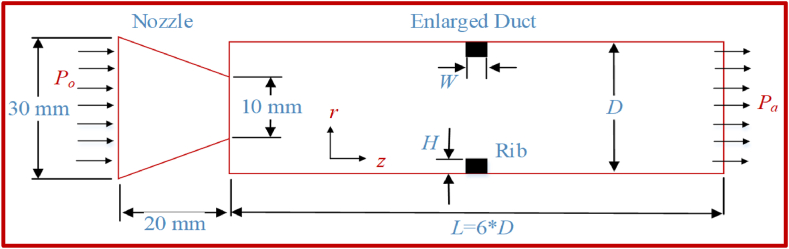
A rectangular rib is placed inside the duct to which the nozzle is attached.

The physical model of the converging nozzle and duct with rib is shown in Fig. 1. The ribs in this study have a 3 mm width, 1 mm–5 mm height, 30 mm nozzle inlet diameter, 10 mm outer diameter, 150 mm duct length, 25 mm duct diameter, and 6.25 model area ratio. The nozzle's flow expands into and through the duct. The flow undergoes several compression and expansion stages due to the expansion fan before reaching atmospheric pressure at the outlet.

The following assumptions are considered to illustrate the flow inside the duct:

i. The flow is assumed to be steady-state and 2D due to the axisymmetric flow through the nozzle and duct.
ii. Given the considerable turbulent viscous dissipation effects, the turbulent flow is considered.
iii. The fluid is compressible, and its viscosity is a function of temperature.
iv. The flows exit from the duct at atmospheric pressure.

Khan and Aabid [11] employed the standard k-ε turbulence model to simulate the internal flow, as this model gives reasonably good results. Hence, we have used the standard k-ε turbulence model for our problem.

The 2D cylindrical coordinate system's Continuity Equation (1) for compressible flow (based on density) with the steady-state condition is formulated as

(1) $$\frac{1}{r_{d}}\frac{\partial \left(\rho_{a}r_{d}u_{a}\right)}{\partial r_{d}}+\frac{\partial \left(\rho_{a}v_{a}\right)}{\partial z_{d}}=0$$

The flow velocity $u_{a}$ is given by Equation (2) as the z-momentum equation for the time-averaged velocity

(2) $$\frac{1}{r_{d}}\frac{\partial \left(\rho_{a}r_{d}u_{a}u_{a}\right)}{\partial z_{d}}+\frac{1}{r_{d}}\frac{\partial \left(\rho_{a}v_{a}u_{a}\right)}{\partial r_{d}}=-\frac{\partial p}{\partial r_{d}}+\left(\mu^{\prime }+\mu_{t}^{\prime }\right)\frac{\partial }{\partial z_{d}}\left[2\frac{\partial u_{a}}{\partial z_{d}}-\frac{2}{3}\left(\nabla .\vec{v_{a}}\right)\right]+\left(\mu^{\prime }+\mu_{t}^{\prime }\right)\frac{\partial }{\partial r_{d}}\left[\frac{\partial u_{a}}{\partial r_{d}}+\frac{\partial v_{a}}{\partial z_{d}}\right]$$

The respective velocity $v_{a}$ is represented by Equation (3) for the *r*-momentum radial velocity:

(3) $$\frac{1}{r_{d}}\frac{\partial \left(\rho_{a}r_{d}u_{a}v_{a}\right)}{\partial z_{d}}+\frac{1}{r_{d}}\frac{\partial \left(\rho_{a}v_{a}v_{a}\right)}{\partial r_{d}}=-\frac{\partial p}{\partial r_{d}}+\left(\mu^{\prime }+\mu_{t}^{\prime }\right)\frac{\partial }{\partial r_{d}}\left[\left(2\frac{\partial u_{a}}{\partial z_{d}}-\frac{2}{3}\left(\nabla .\vec{v_{a}}\right)\right)\right]+\left(\mu^{\prime }+\mu_{t}^{\prime }\right)\frac{\partial }{\partial z_{d}}\left[\left(\frac{\partial u_{a}}{\partial r_{d}}+\frac{\partial v_{a}}{\partial z_{d}}\right)\right]-2\frac{\left(\mu^{\prime }+{\mu_{t}}^{\prime }\right)v}{{r_{d}}^{2}}+\frac{2}{3}\frac{1}{r_{d}}\left(\mu^{\prime }+\mu_{t}^{\prime }\right)\left(\nabla .\vec{v_{a}}\right)$$

The term $\vec{v}$ in Equations (2), (3)) is given by (Equation (4))

(4) $$\nabla .\vec{v_{a}}=\frac{\partial u_{a}}{\partial z_{d}}+\frac{\partial v_{a}}{\partial r_{d}}+\frac{v_{a}}{r_{d}}$$

According to the governing equations of the turbulent flow regime, $\mu^{\prime }+\mu_{t}^{\prime }$ is the effective viscosity. Since viscosity is a function of temperature Ta, the gas density is used as an ideal gas constant in the compressible flow analysis. Sutherland's three-coefficient viscosity model is represented as:

(5) $$\mu^{\prime }=\mu_{o}^{\prime }{\left(\frac{T_{a}}{T_{a,o}}\right)}^{3/2}\frac{T_{a,o}+S^{\prime }}{T_{a}+S^{\prime }}$$

A reference viscosity value in kg/m-s is represented as $\mu^{\prime }$_*o*_, where $\mu^{\prime }$ is the value of the viscosity as mentioned in Equation (5). *T*_*a*_ represents static temperature, *K* represents the temperature of a standard reference, and $S^{\prime }$ stands for the temperature-dependent Sutherland constant. The flow field temperature is obtained using Equation (6), the energy equation.

(6) $$\frac{\partial \left(\rho_{a}u_{a}T_{a}\right)}{\partial z_{d}}+\frac{1}{r_{d}}\frac{\partial \left(\rho_{a}v_{a}T_{a}\right)}{\partial r_{d}}=\frac{\partial }{\partial z_{d}}\left[\left(\frac{k}{{C_{p}}^{\prime }}+\frac{{\mu_{t}}^{\prime }}{{\mathrm{Pr}}_{t}}\right)\frac{\partial T_{a}}{\partial z_{d}}\right]+\frac{1}{r_{d}}\frac{\partial }{\partial r_{d}}\left[r_{d}\left(\frac{k}{{C_{p}}^{\prime }}+\frac{{\mu_{t}}^{\prime }}{{\mathrm{Pr}}_{t}}\right)\frac{\partial T_{a}}{\partial r_{d}}\right]$$

The quantities mentioned in the above Equation (6) are as follows:

• The term represents the fluid thermophysical property $\left(\frac{k}{{C_{p}}^{\prime }}+\frac{{\mu_{t}}^{\prime }}{\bar{{\mathrm{Pr}}_{t}}}\right)$.
• Thermal conductivity is *k* in W/m^2^ k.
• The specific heat capacity in kJ/kg-K is denoted as ${C_{p}}^{\prime }$.
• The turbulent viscosity is given by ${\mu_{t}}^{\prime }$ in kg/m-s.
• The term gives the turbulent Prandtl number $\bar{{\mathrm{Pr}}_{t}}$.

Because of its economy, durability, and sufficient accuracy, the k-ε turbulence model is widely employed in various flow simulations. The ANSYS Fluent software includes the k-turbulence model used in this study. Because of the K-equation, we can calculate the turbulent kinetic energy (Equation (6)).

(7) $$\frac{\partial \left(\rho_{a}u_{a}K\right)}{\partial z_{d}}+\frac{1}{r_{d}}\frac{\partial \left(\rho_{a}v_{a}K\right)}{\partial r_{d}}=\frac{\partial }{\partial z_{d}}\left[\left(\mu^{\prime }+\frac{{\mu_{t}}^{\prime }}{\sigma_{k}}\right)\frac{\partial K}{\partial z_{d}}\right]+\frac{1}{r_{d}}\frac{\partial }{\partial r_{d}}\left[r_{d}\left(\mu^{\prime }+\frac{{\mu_{t}}^{\prime }}{\sigma_{k}}\right)\frac{\partial K}{\partial r_{d}}\right]-\rho_{a}\varepsilon +M_{X}$$

The term *M*_*X*_ is the turbulence generation given by Equation (8), and the turbulence model constant is $\sigma_{k}$ is associated with the dissipation term, turbulent kinetic energy dissipation rate is represented by ε.

(8) $$M_{X}={\mu_{t}}^{\prime }\left(\frac{\partial u_{a,i}}{\partial x_{a,j}}+\frac{\partial u_{a,j}}{\partial x_{a,i}}\right)\frac{\partial {u\;}_{a,i}}{\partial x_{a,j}}-\frac{2}{3}K\delta_{ij}\frac{\partial u_{a,i}}{\partial x_{a,j}}$$

The kinetic energy of turbulence dissipation (i.e., ε-Equation) is given by

(9) $$\frac{\partial \left(\rho_{a}u_{a}\varepsilon \right)}{\partial z_{d}}+\frac{1}{r_{d}}\frac{\partial \left(\rho_{a}v_{a}\varepsilon \right)}{\partial r_{d}}=\frac{\partial }{\partial z_{d}}\left[\left(\mu^{\prime }+\frac{{\mu_{t}}^{\prime }}{\sigma_{\varepsilon }}\right)\frac{\partial \varepsilon }{\partial z_{d}}\right]+\frac{1}{r_{d}}\frac{\partial }{\partial r_{d}}\left[r_{d}\left(\mu^{\prime }+\frac{{\mu_{t}}^{\prime }}{\sigma_{\varepsilon }}\right)\frac{\partial \varepsilon }{\partial r_{d}}\right]-\bar{C_{1}}\bar{f_{1}}\left(\frac{\varepsilon }{k_{a}}\right)M_{X}-\bar{C_{2}}\bar{f_{2}}\left(\frac{\varepsilon^{2}}{k_{a}}\right)$$

In Equation (9) where ${\mu_{t}}^{\prime }=\rho_{a}\bar{f_{\mu }}\bar{C_{\mu }}{k_{a}}^{2}/\varepsilon$ denotes turbulent viscosity, and the arbitrary constants are denoted as $\bar{C_{\mu }}$, $\bar{C_{1}}$, $\bar{C_{2}}$, $\bar{f_{\mu }}$, $\sigma_{k}$, and $\sigma_{\varepsilon }$ .

The finite volume method is adopted to solve the governing equations of turbulent fluid flow. Given that the flow is purely symmetric, only the upper half of the 2D domain is discretized to save computational time. The second-order accurate implicit scheme is applied to discretize the Equation over the fluid domain. The second-order upwind method is adopted, and the turbulent kinetic energy and dissipation rate equations are discretized.

**Fig. 2:**
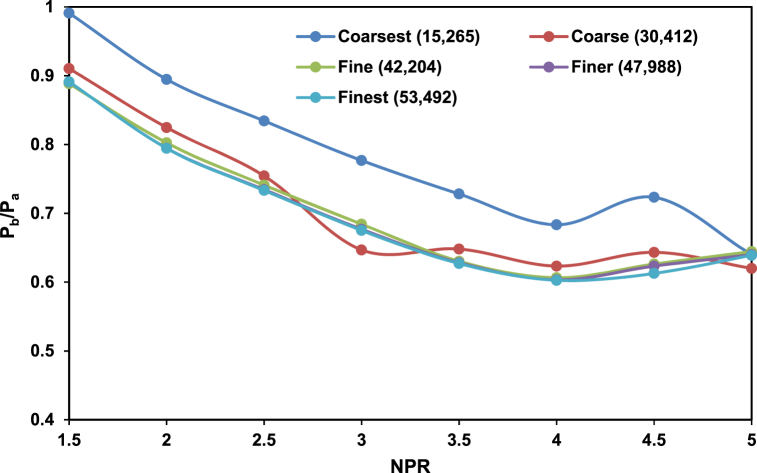
Mesh independence test with different mesh element sizes adopted in the study.

The simulation was performed using ANSYS. The rectangular structured mesh is used to capture the velocity and pressure gradients. Mesh independence test is conducted for coarsest, coarse, fine, more refined, and most refined with their respective element sizes, as shown in Fig. 2. The fine mesh is chosen as further increases in the mesh element sizes provide a similar result. The y + value is 2.919 × 10^−2,^ and the element size is 0.0008. The residuals for the energy and continuity equations are set to 10e^−5^.

## 3 Comparison with previous experimental work

**Fig. 3:**
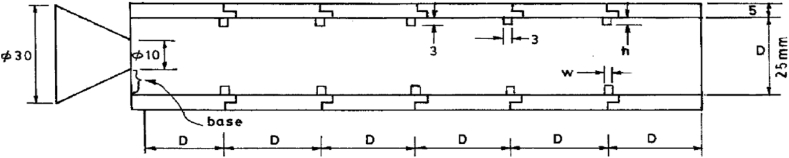
A duct with five ribs was used in the experimental study of Radhakrishnan [11].

The setup of a single duct at different locations was initially compared and validated based on the findings of Rathakrishnan [11], who placed five ribs in a central space in the circular tube, as shown in Fig. 3. Initially, the diameter of the duct was considered to be 25 mm, with an area ratio of 6.25. All measurements were completed with one aspect ratio (width/height, i.e., w/h) of the ribs, and the annular rib heights followed the desired aspect ratio. In the experimental study [11], three aspect ratios, 3:3, 3:2, and 3:1, were tested and simulated. The other parameters of the present investigation were the area ratio, the L/D ratio, and the primary pressure ratio P_0_/P_a_. NPR was considered 2.458, and experiments were done for the same NPR. The L/D ratio ranges from 3 to 6. The base pressure variations with different NPRs and duct lengths for both the conditions of *having* and *not having ribs*; are obtained.

**Fig. 4:**
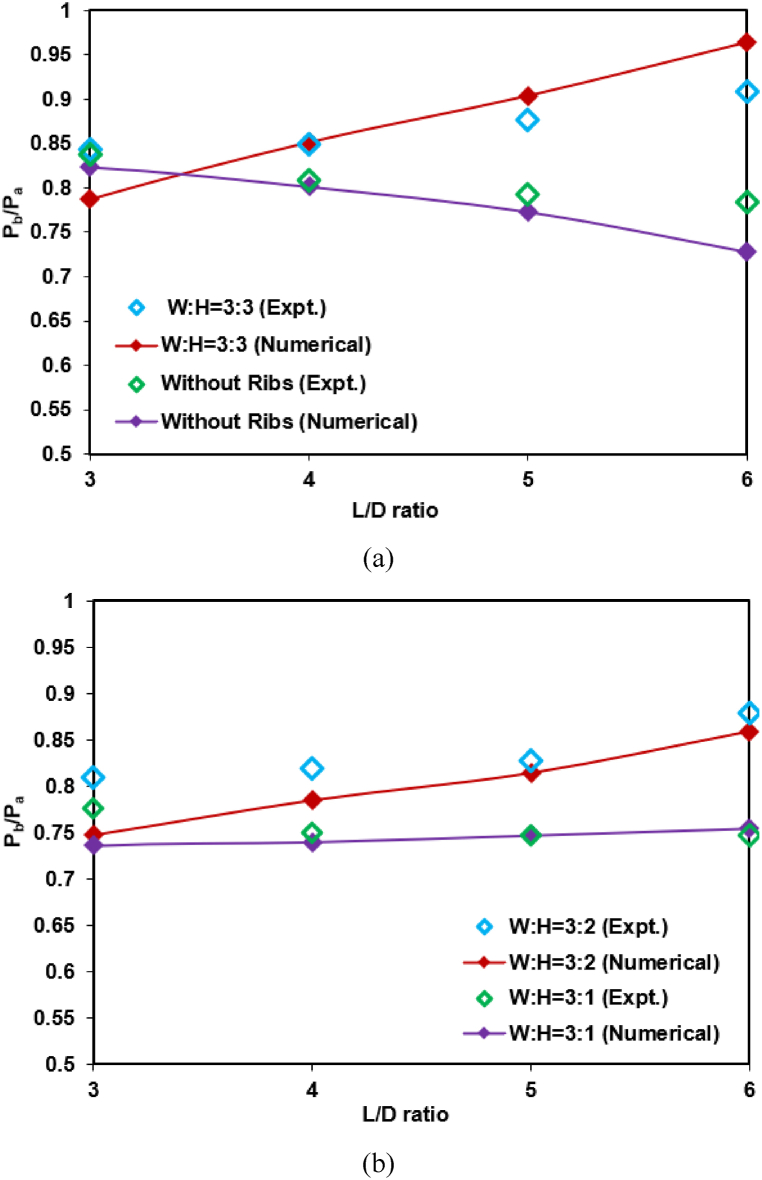
The comparison between the work of Rathakrishnan [19] (experimental work) and present work (numerical results) for a) 3:3 aspect ratio and b) 3:1 and 3:2 aspect ratios.

Fig. 4 (a) and (b) compare the base pressure variations obtained in this work and Rathakrishnan [11]. Fig. 4 (a) compares the base pressure for a rib aspect ratio of 3:3, whereas Fig. 4 (b) compares the simulation and the experimental results for a rib aspect ratio of 3:1 and 3:2. Nevertheless, the numerical results from this work follow a similar trend to the experimental data of Rathakrishnan [11]. The jet will be divided into two streams when the flow exits the nozzle. One is the main jet, and the other is a wake zone where the low-pressure region is created. After the dividing streamline is reattached, a boundary layer will be formed. Depending on the rib location and the boundary layer, there will be backflow towards the base region. The ambient pressure will influence lower L/D ratios flow inside the duct, and the same may not be true when the L/D = 6. This may be the reason for the variations in the base pressure.

## 4 Influence of single, multiple, and without ribs on base pressure

**Fig. 5:**
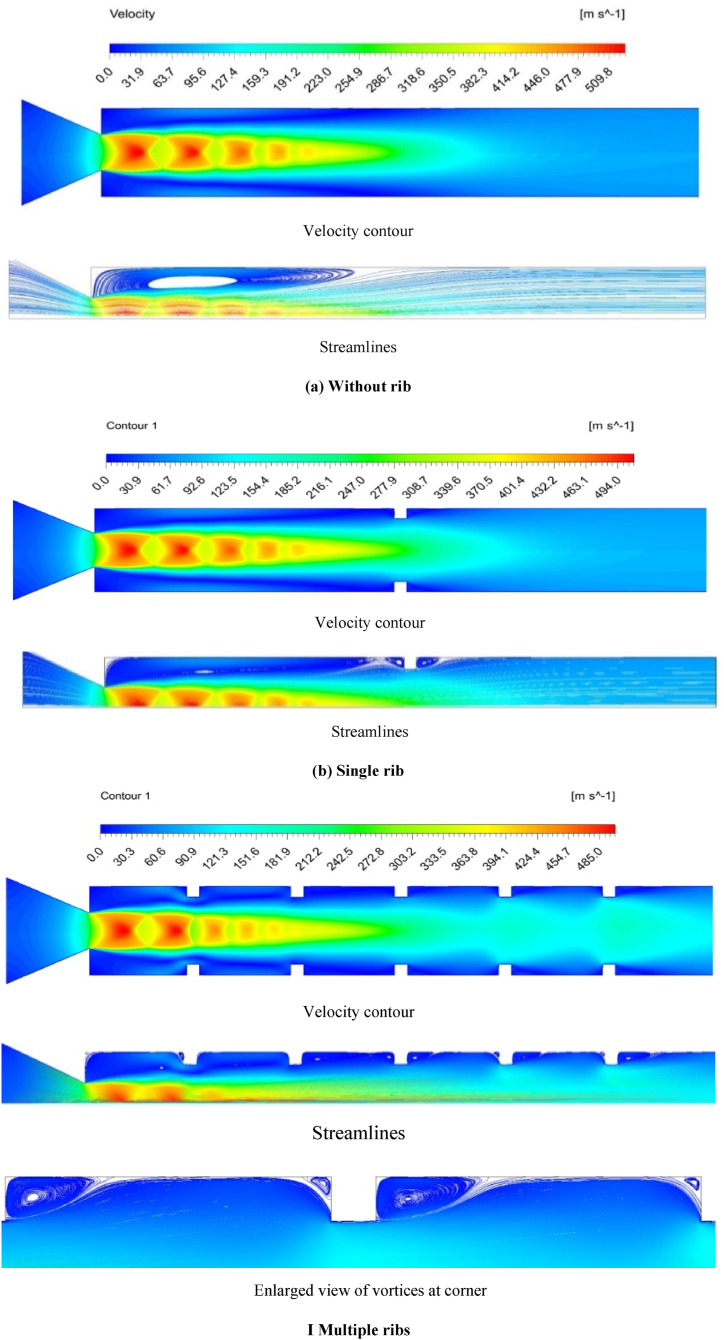
Velocity contours, streamlines, and view of recirculation zones of the flow field in the (a) duct without ribs, (b) duct with a single rib, and (c) duct with multiple ribs.

Fig. 5 (a) to (c) compare the flow field distributions in the duct with single, multiple, and no ribs. The duct wall is smooth without the presence of any rib. For the enlarged duct with a single rib, the rib is placed in the 3D position. In contrast, each rib is placed at the 1D position away from one another along the duct. The comparison of velocity contours and streamlines reveals that the recirculation zone and velocity variation can be ascribed to the sudden flow expansion from the converging nozzle. The flow undergoes sonic conditions at the nozzle exit under a primary pressure ratio 1.89. The primary pressure for which the experiments and simulations are conducted and compared at NPR, i.e. $\frac{P_{0}}{P_{a}}=2.458$. For NPR above 1.89, an expansion fan is observed at the nozzle exit across all cases, and the shear layer passes through the expansion fan. An expansion fan's presence decreases the base pressure and increases the reattachment length between the 3D and 4D positions. When multiple ribs are placed 1D apart, the flow field is disturbed, and the reattachment length is not observed, as shown in Fig. 5(c). The recirculation zones and reattachment length are visible in cases of a single ribbed duct and no rib duct. In all instances, the velocity contour shows variations in the velocity field along the duct length away from the nozzle exit.

Due to the presence of the rib, flow initially gets compressed and expanded during the expansion process in the enlarged duct. Due to the presence of an expansion fan, diamond-shaped shock waves can be seen at the nozzle exit. At distances beyond 3D, the velocity field becomes more uniform and achieves a constant and consistent magnitude from that point. In a single rib, three vortices are observed, one at the base and two at the rib corners. These secondary vortices are caused by the flow trapped at the wake region of the duct and generate the backward flow of fluid toward the primary vortex (i.e., in the base corner). Fig. 5(b) shows that the maximum velocity zone slightly extends downstream of the duct due to a rib's presence, reducing the flow area and restricting the flow expansion. When multiple annular ribs are attached, as shown in Fig. 5(c), the velocity increases up to the end of the duct due to the ribs' restrictions. The flow-through multiple ribbed geometries are identified by forming numerous secondary vortices around the rib corners and modifying primary vortices.

**Fig. 6:**
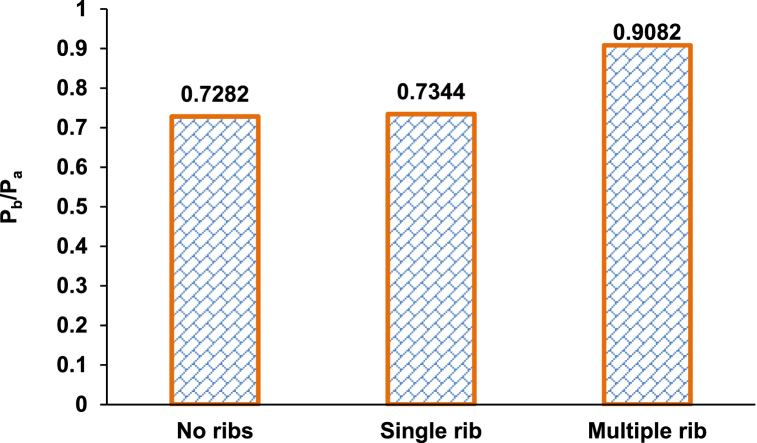
Variations in non-dimensional base pressure with ribs (aspect ratio = 3:3 and NPR = 2.458).

The base pressure values obtained from these different configurations are plotted in Fig. 6. The lowest and highest base pressures are observed in no rib ducts and multiple ribs, respectively. Meanwhile, using a single rib moderately increases the base pressure, which can be attributed to the formation of primary and secondary vortices and the variations in the flow field. Flow reversal becomes more evident in multiple ribbed ducts as the flow is disturbed from the 1D to 5D rib positions. In a single rib case, the flow expands in the duct, and the disturbance to the flow is relatively minimal due to the insufficient height of the rib. This study provides further insights into the effect of rib aspect ratio and NPR when considering a single rib, whereas Rathakrishnan [11] thought of multiple ribs. The presence of five ribs in the duct will form many secondary vortices at sharp corners, and their interaction will make flow oscillatory. The main issue in using multiple ribs is the significant variation in wall pressure and the flow field, which triggers oscillations in the duct's flow field. Numerous ribs can also produce additional frictional drag, and the boundary layer undergoes many interactions with the ribs and duct wall.

## 5 Effect of aspect ratio and rib location on base pressure

**Fig. 7:**
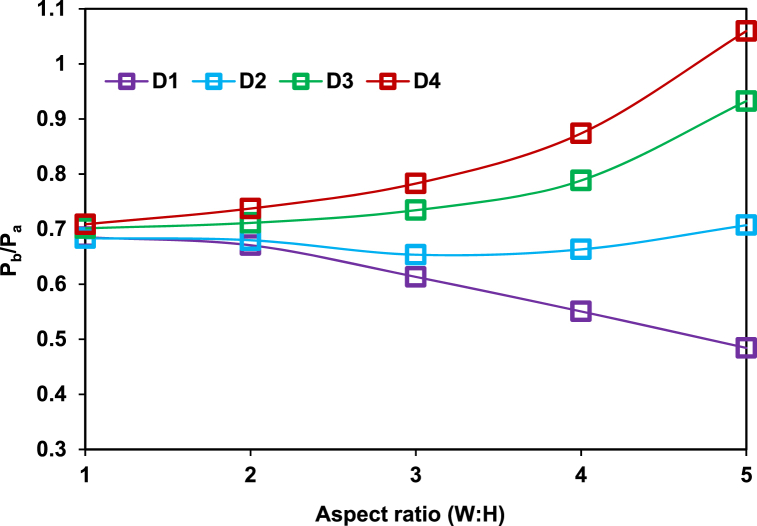
Base pressure for aspect ratio (W: H) at different duct positions.

The rib position (D) varies from 1D to 4D, the rib height ranges from 1 to 5, the width is fixed to 3 mm, resulting in aspect ratios from 3:1 to 3:5, and the maximum duct length is 6D (i.e., L = 150 mm). The base pressure is obtained from the numerical analysis, and the results are plotted against the aspect ratio (W: H) for different duct positions, as shown in Fig. 7. At the 1D location of the annular rib, changing the rib height from 1 to 5 increases the base pressure compared with the duct with no rib, negatively affecting the base drag. For the 2D rib position, the base pressure remains nearly the same, with a slight increase in the rib's height of 5 mm at the end. For rib positions 3D and 4D, the base pressure linearly increases along with the rib height and reaches its maximum at the 5 mm rib height.

The shear layer expands and attaches to the downstream duct walls as the flow exits from the nozzle. The reattachment point depends on the level of expansion and the inertia level at the nozzle exit. The vortex is formed as the flow separation at the nozzle exit influences and regulates the base pressure. The base pressure controlled by the primary vortex at the nozzle base depends on the flow Mach number at the nozzle exit and the reattachment length. The low-pressure level at the base region relies on the strength of the primary vortex. Given this low-pressure zone, the fluid from the duct wall moves downstream of the flow, and the reattachment point moves back to the base region. This extra mass reenters the base region and is ejected to the main flow stream through the shear layer entrainment. This cycle of mass backflow and mass reinjection into the main flow continues steadily. Wick [5] described this phenomenon as a jet pump action that reduces the primary vortex's strength and increases the base pressure to a level higher than obtained without the backflow of fluid from the reattachment point. This phenomenon is related to the sudden expansion in an enlarged duct without a rib (i.e., for the smooth wall).

**Fig. 8:**
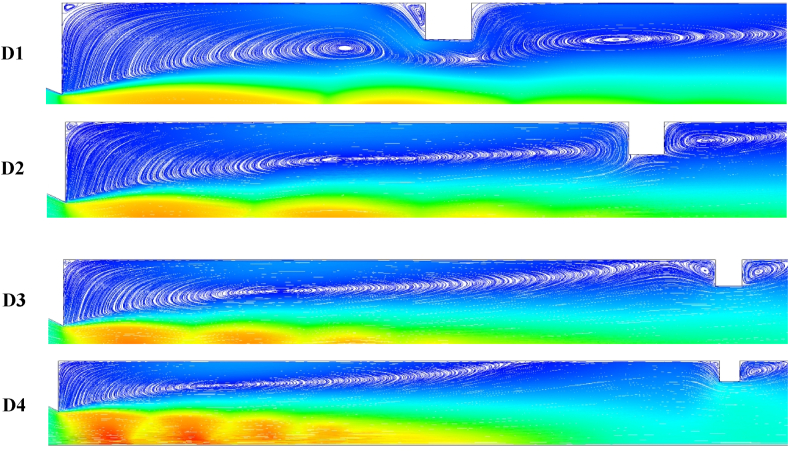
Streamlines showing recirculation zones.

Depending on the rib height during the expansion of the boundary layer in the duct with ribs, the backflow towards the primary vortex from the reattached boundary layer can be prevented. The expanded flow field is disturbed, and passive rib control becomes effective. The reverse flow is partially blocked when the rib height is 1 mm or 2 mm. Given the presence of a rib, an expansion fan is created due to the NPR being more than 1.89 required for correct expansion. The expansion of the flow is observed while the jet passes through the expansion fan. A low base pressure region is formed near the main flow below the boundary layer. The interaction among the expansion waves, compression waves, and reverse flow creates a low-pressure area. The strength of the low-pressure region depends on the location of the rib inside the duct, as shown in Fig. 8, when the rib height is 1 mm or 2 mm and when the base pressure increases. The vortices’ highest and lowest strengths are recorded at the 4D and 1D positions. At the latter, the wave interaction with the reverse flow and the secondary vortex enters a relaxation area outside the reattachment point, thereby increasing the base pressure. However, the relaxation area is restricted at the 1D position near the base, creating a low base pressure region. Across different places, the backward fluid flow increases at high aspect ratios of 3:4 and 3:5. In contrast; the flow does not move back to the base region at an aspect ratio of 3:1 to 3:2. This case of rib location and geometry increases the base suction.

**Fig. 9:**
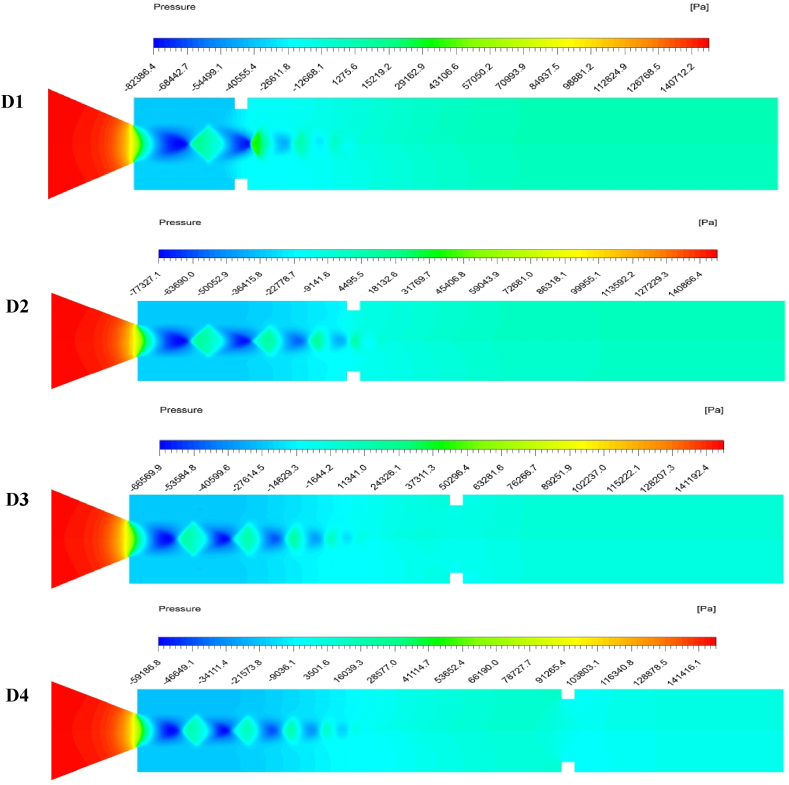
The contour of pressure distribution when ribs are located at different distances from the nozzle exit.

Backward fluid flow is blocked at a rib aspect ratio of 3:3 to 3:5. The lowest and highest base pressures are recorded at the 1D and 4D positions, respectively, as shown in Fig. 9. The increase or decrease in base pressure depends on the availability of a relaxation area. The numerical values of base pressure for aspect ratios 3:4 and 3:5 at the 1D and 2D positions are lower than those for aspect ratios 3:1 and 3:2 at the exact locations. For aspect ratios 3:4 and 3:5, the reverse flow is blocked, and no interactions occur with the reverse flow. In a few, the interface only occurs between the waves and secondary vortices. In such a case, the secondary vortex is more significant than that generated at 1 mm or 2 mm height, resulting in a low base pressure. At 3D and 4D rib positions, the base pressure for a rib height of 4 mm or 5 mm is higher than that for a rib height of 1 mm or 2 mm because the flow in the former is blocked. When rib heights are 4 and 5 mm, relaxation available to the flow is relatively small, and vortices formed at the sharp corners interact with the base vortex. This interaction of secondary vortices will increase the base pressure.

The rib height of 3 mm neither allows nor blocks the backward flow. Fig. 8 presents the vortices formed around the rib corners. Only one primary vortex is developed for a smooth duct, as shown in Fig. 5 (a). Numerous vortices are created in the wake corner, and the primary vortex is divided due to the fluid entrapment around the corners. At the 1D position (Fig. 8), the reattachment point is far from the 1D location, splitting the primary vortex. In contrast, the secondary vortex is connected to the primary vortex with less backflow mass. A closer look at the 1D position vortex reveals that the flow cannot locate the rib given its relative distance from the base region, creating a powerful vortex and a low base pressure region.

For the 4D position, the rib lies downstream of the reattachment point, allows an increased backward flow of fluid, and weakens the vortex at the base, as shown in Fig. 8. The position and height of the rib significantly affect the base pressure values. Lower rib heights are recommended for nearer locations, whereas higher rib heights are proposed for 3D and 4D locations.

Fig. 9 shows the variations in base pressure along the duct as the position of the 3:3 rib aspect ratio changes from 1D to 4D. The NPR and duct diameter are fixed at 2.458 and 25 mm, respectively. The contours of pressure with the rib location indicate the flow characteristics. Low base pressure is observed at the nozzle exit at the 1D position, whereas an increased base pressure is reported at the 4D location. The pressure contour indicates higher pressures dominate the duct and movement of the rib downstream of the reattachment point. A diamond-shaped lower-pressure region is observed along the axial direction of the nozzle exit.

Meanwhile, small higher-pressure regions are observed at the 1D position, but as the rib location shifts downstream, these regions become more extensive and energetic. The location and distance between these pressure regions depend on the rib positions. The expansion fan from the nozzle lip along the circumferential direction compresses the flow and subsequently forms a low-pressure area. Due to their continuous expansion, the flow reattaches itself to the wall and undergoes compression, forming a higher-pressure region. This alternate expansion and compression process create diamond shapes. As the flow travels axially, the velocity magnitude reduces due to shear and eddy losses, promoting uniform pressure distribution in the duct.

## 6 NPR and rib size affecting base pressure

**Fig. 10:**
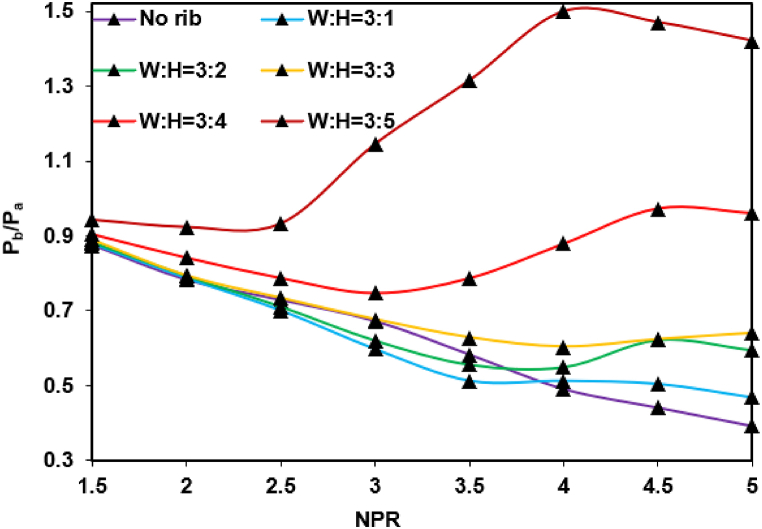
Changes in base pressure for a single rib with aspect ratios of 3:1 to 3:5 and NPR of 1.5–5 compared with that for no rib duct.

Fig. 10 shows the passive effect of control on the ribs with NPR, varying from 1.5 to 5. The rib size (i.e., aspect ratio) continuously increases from 3:1 to 3:5, whereas the rib position is fixed at 3D. Increasing NPR will reduce the base pressure for the smooth duct without control. For the converging nozzle that can generate only a sonic Mach number, an NPR of 1.89 is needed to obtain a sonic Mach number and is under-expanded for an NPR of more than 1.89. Therefore, the flow undergoes the correct expansion stages and under-expansion when the NPR increases from 1.5 to 5. When the NPR exceeds 1.89, an expansion fan is formed at the nozzle exit, and the shear layer moves through the expansion fan, thereby resulting in the expansion of the jet. Given an expansion fan's presence, the base pressure assumes low values, thereby considerably increasing the reattachment length compared to when the NPR is less than 2. The dependence of the reattachment point concerning NPR is irrespective of the type of control employed. The reattachment length directly depends on the NPR and increases along with an increase in the NPR. A low NPR corresponds to a short reattachment length and small vortices at the base zone, promoting the reverse flow of mass ejected to the mainstream flow back through the shear layer. As shown in Fig. 10, the same flow physics agrees with the flow without rib, given that the base pressure continuously decreases along with an increasing reattachment length. With the presence of a rib, the flow downstream of the reattachment point faces additional barriers as the rib height increases. Because of this, the flow from the converging nozzle discharged into the enlarged duct tends to attach with reattachment lengths that are not optimal for an active vortex in the base area. NPR has a negligible effect on the base pressure at high area ratios due to this process [26]. Fig. 10 shows that the base pressure neither decreases nor increases when the annular rib is attached to the 3D position and when the NPR increases. A closer inspection of this figure highlights the effectiveness of the control in increasing the base pressure at the early NPR levels, along with an increasing aspect ratio.

The discrepancies in the results can be attributed to the blockage caused by the ribs. The other factors are its sharp corner effects, the rib with the shear layer, and waves forming at different NPRs. The growth in pressure in the wake of a progressive increase in NPR can be understood as increasing mass flows toward the base region in a given time. However, the reversal of control at a high NPR needs further exploration. The level of under-expansion increases with an increase in NPR, developing the shear layer for considerable distances and causing a more significant area for the vortex at the base. Since the rib is at the 3D position, shifting the reattachment point to NPR will reduce the fluid backflow. However, the base pressure is 250 % greater than the base pressure in the duct without a rib.

**Fig. 11:**
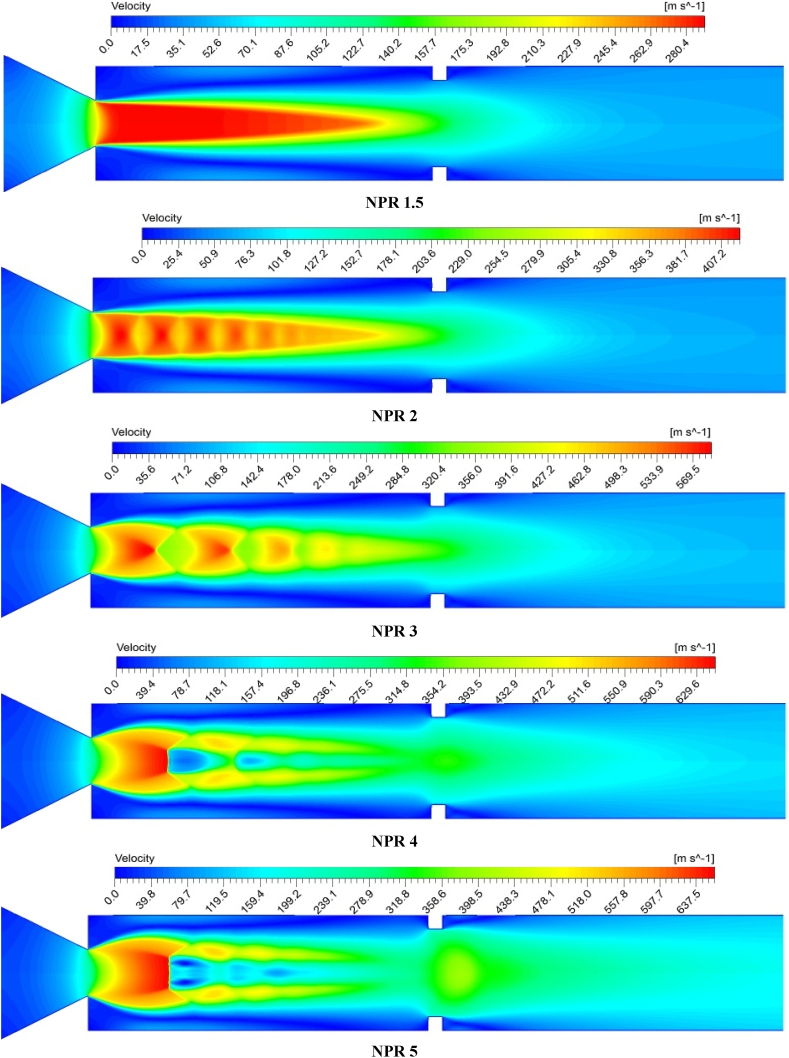
Changes in flow velocity pattern increase with NPR, particularly at the rib position with W:H = 3:3.

Fig. 11 shows the velocity field pattern at an NPR ranging from 1.5 to 5, the aspect ratio of 3:3, and the 3D position. NPR = 1.5 belongs to the condition where the nozzle is yet to reach critical condition. The maximum velocity zone resembles the pattern of a flame coming out of a candle or lamp. The NPR increases from 2 to 5; the expansion fan becomes visible at the nozzle exit. At an NPR of 2, the waves are weak yet visible when the flow undergoes alternate compression and expansion cycles. At an NPR of 3, the expansion fan has increased in size and is located at a significant axial distance from the nozzle exit. A high under-expansion level at an NPR of 4 creates an expansion wave as the rib size increases. At an NPR of 5, the wave becomes more powerful and undergoes the expansion process. In contrast, the fluid is slightly recompressed at the rib location and is ejected along with high velocity, as shown in Fig. 11. The flow velocity at the duct exit position is several times higher than that at a low NPR.

**Fig. 12:**
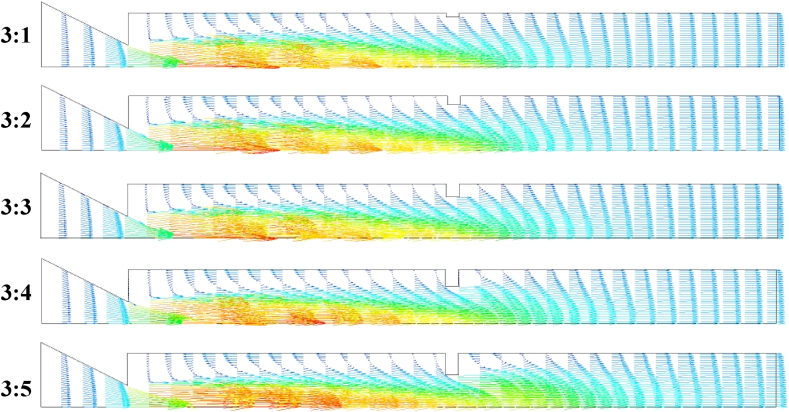
Influence on flow field velocity vectors at different aspect ratios and NPR = 2.5.

Fig. 12 shows the velocity vectors in the duct formed for aspect ratios of 3:1 to 3:5, and NPR kept constant at 2.5. The vectors are entrained at the rib corners that block the flow along the duct. The magnitude of vectors is kept constant for all aspects of flow separation, reversal, and primary and secondary vortex strengths. Increasing the rib height at an NPR of 2.5 leads to the reverse of more mass fluid into the primary vortex region. Consequently, the size of the secondary and ternary vortices at the rib corner downstream of the duct also increases and recirculates a large fluid. Green arrows denote the flow velocity vectors along the shear layer where the flow separates. For those ribs with an aspect ratio of 3:5, the flow is recompressed at the rib location. Later, it is ejected at a high velocity. The vectors immediately become uniform at a low rib aspect ratio. The flow does not settle soon after the rib for high aspect ratios, given that the boundary layer has sufficiently shifted downstream. It achieves a high-velocity magnitude at the axial position at the duct exit. The first wave suddenly faces a high-pressure region as the flow velocity is reduced at the center. Nevertheless, the flow velocity adjusts in the shear and recirculation zones.

**Fig. 13:**
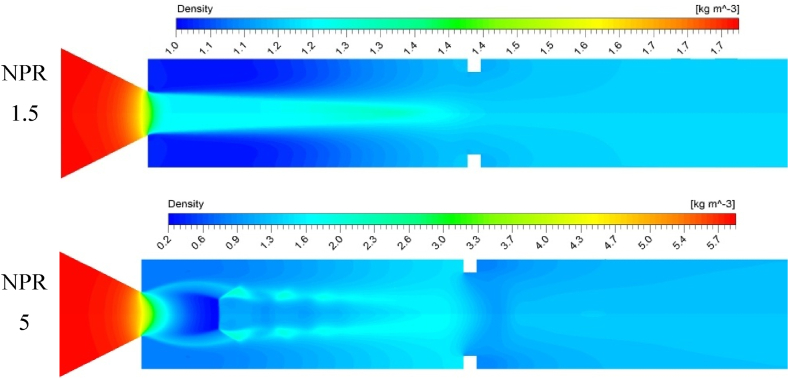
Compressed air zones and outline of density variation with NPR.

Fig. 13 presents the different density zones for the compression shear flow in the form of the density contours at NPRs of 1.5 and 5. The rib is placed at a 3D position when the rib height is 3 mm. The compressed fluid is found in a converging nozzle part for all cases irrespective of the expansion level (i.e., NPR) and inertia level (i.e., Mach number). Given the pressure differences, the flow expands from the nozzle to the duct, where the flow experiences adverse pressure gradients, thereby expanding and compressing the air. The density is uniform and low at the base region at a low NPR. By contrast, a higher mass flow reversal is observed at a high NPR and in the presence of a rib in the duct, thereby marginally increasing the density and base pressure. Here, the expansion is 2.5 times stronger at an NPR of 5, suggesting that the flow compression is located when the velocity reaches the maximum value. In other words, the flow has expanded in these regions, reducing the pressure and rapidly accumulating flow particles at a given period and controlling volume. The significance of the density contour can be determined at NPR of 5, where the density is diminished and attains a bare minimum just after the nozzle exit due to the jet's expansion.

**Fig. 14:**
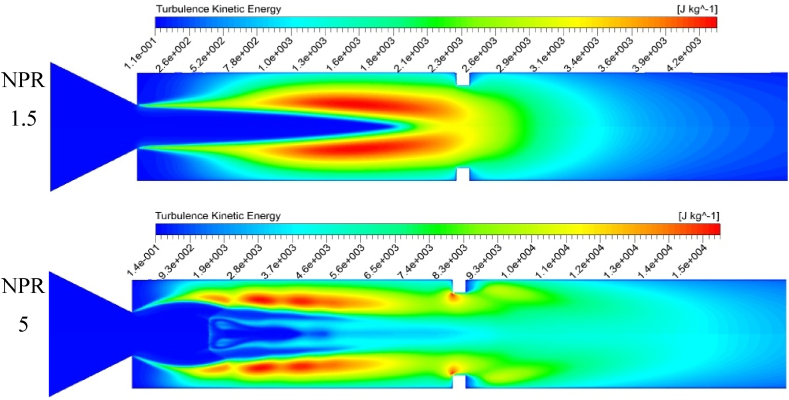
Variations in turbulent kinetic energy obtained from the k-equation model.

**Fig. 15:**
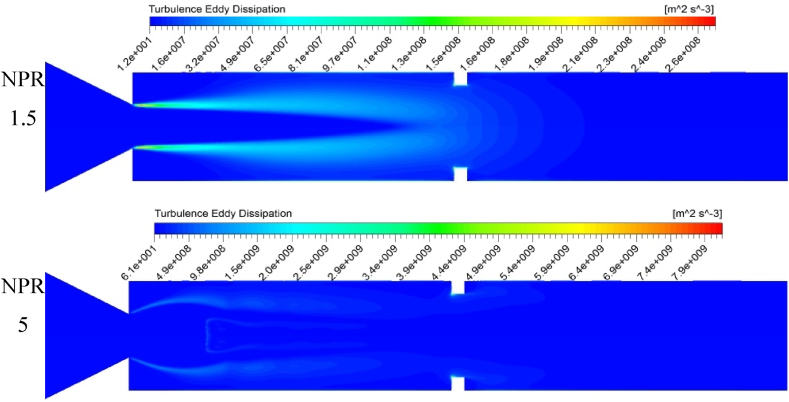
Turbulence eddy dissipation with an increasing NPR.

Fig. 14 presents the duct's turbulence kinetic energy (TKE) distribution with a rib at the 3D position and an NPR of 1.5 and 5. The flow and velocity vectors are straight along the flow direction in the smooth duct with no rib. The TKE is also very low in ducts without ribs. The presence of a rib leads to the extreme creation of TKE due to turbulent eddies in the tube and compression side of the rib, as shown in Fig. 14, Fig. 15. The highest TKE at the ribbed wall is remarkably higher than that in a smooth duct wall. As the arrows in Fig. 12 indicate, the flow velocity vectors move toward the rib and duct wall's side surfaces with a high TKE. After the flow particles reach the eddy region near the rib corners, the particles are entrained to the rib and wall's windward surfaces.

Fig. 14 illustrates the turbulent eddy dissipation for NPRs 1.5 and 5 with a rib at the 3D position for an aspect ratio of 3:3. The eddy dissipation is seen at the shear layer, where the flow separates. The eddies are broader and more prolonged at an NPR of 1.5 as the shear layer increases along with a low level of expansion. At an NPR of 5, a high expansion level is observed, thereby creating sharp eddies at the shear layer. The vortices are noticeable at the nozzle (nozzle lip), and the rib corner at these regions rotates fluid particles and triggers sizeable viscous dissipations and shear losses.

## 7 Effect of area ratio on base pressure

**Fig. 16:**
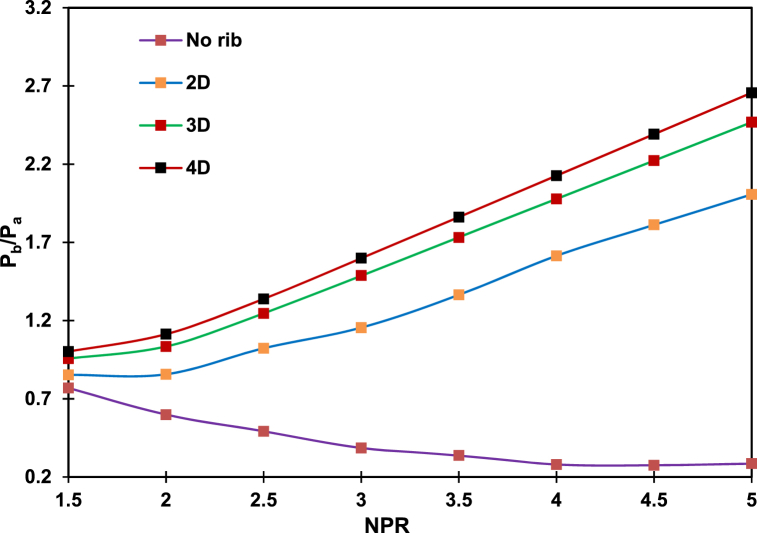
Base pressure changes for 1.5 to 5 NPR, 2D to 4D rib positions, 3:3 aspect ratio, and 18 mm duct diameter.

Fig. 16 presents the changes in base pressure when the duct area ratio is reduced to 18 mm. The aspect ratio of the rib is fixed to 3:3. Decreasing the area ratio reduces the reattachment length. The base pressure decreases with an increasing NPR even though the jets are under-expanded for the no-rib case.

Regarding the ribbed duct, the base pressure rises in tandem with NPR. Whenever the nozzle is under-expanded or flowing under the influence of a favorable pressure gradient, the control becomes effective and increases the base pressure. When the rib is at the 2D position, the base pressure significantly and consistently increases compared with a duct without a rib. However, a small increment in base pressure is seen when ribs are at 3D and 4D positions. We first discuss why the base pressure decreases along with an increasing NPR in the duct without ribs.

Increased NPR results in rapid flow expansion in ducts without ribs. As a result of the expansion level (NPR), the shear layer travels further before attaching to the duct wall. TKE dissipation level and viscous flow effects are, therefore, both low. Increasing the distance of the viscous shear layer's distance increases the primary vortex's size. It decreases the amount of fluid reversal since the result is a steadily decreasing base pressure. An expansion fan at the nozzle exit affects the flow when a single rib with a height of 1 mm is located. In addition, as the rib position increases, the flow expansion level aids it in moving toward the reattachment point. There is little difference in base pressure between 3D and 4D rib positions, suggesting that placing the rib at 5D will not significantly impact the base pressure. The most significant change in base pressure is observed at the 2D location for ribs with an aspect ratio of 3:3.

**Fig. 17:**
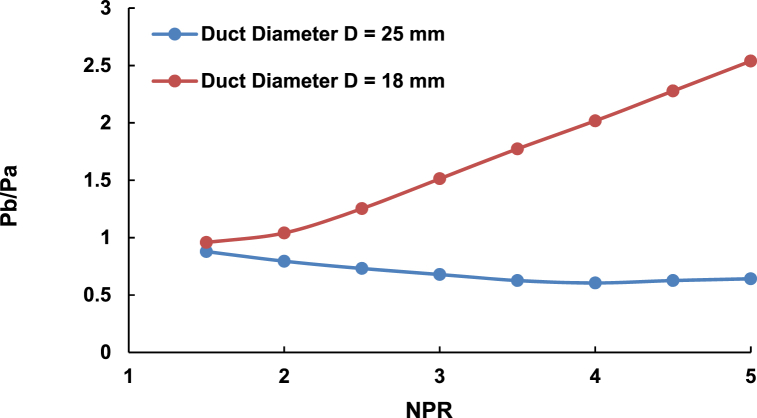
The difference in base pressures for 18 mm and 25 mm duct diameters, fixed 3D position, and 3:3 fixed aspect ratio.

Fig. 17 shows the effect of the area ratio for a fixed 3D rib position, the fixed aspect ratio of 3:3, and duct diameters of 18 mm and 25 mm. The maximum base pressures for these duct diameters are 2.5 and 0.6, with the former almost twice as remarkable as the original base pressure. Such increment can be ascribed to the reattachment length, which is longer for the 25 mm than for the 18 mm duct diameter. As the flow exits the nozzle, a sudden increase in the area leads to a flow expansion. For the 25 mm duct diameter, increasing the NPR from 1.5 to 5 expands the flow as the jet becomes under-expanded at a higher NPR. Therefore, the 3:3 rib aspect ratio cannot influence the flow in the enlarged duct. However, for the 18 mm duct diameter, the duct area decreases by nearly 50 % due to the short reattachment length. These circumstances become advantages in the employment of ribs or any passive control, resulting in a significant increase in the base pressure.

## 8 Conclusions

We may draw the following conclusions based on the above discussions for a suddenly expanded flow from a converging nozzle at a sonic Mach number.

1. There is no need to use five ribs as a passive control mechanism to control the base pressure, as is used in the reference paper. This study suggests that the same results can be achieved by a single rib since an increased number of ribs will increase the total weight of the system and the skin friction drag.
2. Results show that using a single rib too dramatically increases the base pressure for duct 18 mm even for a rib height of 3 mm. However, it is case-sensitive, and one has to find the best combination of the inertia level and geometrical parameters.
3. The simulation results are validated based on the experimental results presented in a previous study. The simulation results show good agreement with the experimental results obtained by Rathakrishnan [11]. Another simulation is also performed for a single rib fixed at the 3D position from the base; as for this duct diameter of 25 mm, the flow is expected to get reattached with the duct wall at 3D. The NPR varies from 1.5 to 5, the duct diameter is selected at 25 mm, and the rib aspect ratios range from 3:1 to 3:5. A duct of 18 mm was chosen to study the impact of lower area ratio on base pressure and the influence of rib under varying ribs locations sizes as for this duct diameter of 18 mm the flow will get reattached with the duct wall between 1D to 2D. The presence of rib generates secondary and tertiary vortices at the rib corners, which interact with the base vortex and significantly change the flow field, increasing the base pressure even for a rib height of 3 mm.
4. Ribs with a high aspect ratio are very useful under a favorable pressure gradient for an area ratio of 6.25 if the mission requirement is to enhance the base pressure values.
5. The results obtained at low area ratios behave differently. Specifically, the base pressure suddenly increases from NPR 3 and above or right from the beginning, suggesting that NPR, rib geometry, and area ratio have certain limiting values to increase base pressure. Moreover, passive control (in the form of ribs) becomes effective whenever the jets are under-expanded.
6. It is important to note that when the relief effect caused by an increased area ratio exceeds a specific limit, flow from the converging nozzle discharged into the enlarged duct tends toward a reattachment length that is not optimal for a strong vortex at its base. At high area ratios, the effect of NPR on base pressure is negligible because of this process.

## Future works

1. The three-dimensional analysis is not performed, and the spanwise flow analysis requires more attention, which is not addressed due to limited experimental results.
2. A large-eddy simulation can be performed for the same case to get an in-depth idea of the flow.
3. Active and passive control can be used to control the base pressure. This hybrid control mechanism may give some exciting results.

## Data availability statement

Data will be made available on reasonable request.

## CRediT authorship contribution statement

**Ambareen Khan:** Writing – original draft, Funding acquisition, Data curation, Conceptualization. **Sher Afghan Khan:** Writing – review & editing, Supervision, Data curation, Conceptualization. **Vijayanandh Raja:** Writing – review & editing, Formal analysis, Conceptualization. **Abdul Aabid:** Writing – review & editing, Formal analysis, Data curation. **Muneer Baig:** Funding acquisition, Formal analysis, Data curation.

## Declaration of competing interest

The authors declare the following financial interests/personal relationships which may be considered as potential competing interests.
